# The comparison of remimazolam and midazolam in bronchoscopic sedation: a systematic review and meta-analysis of randomized controlled trials

**DOI:** 10.3389/fmed.2025.1581280

**Published:** 2025-07-08

**Authors:** Yahya Kayed AbuJwaid, Salahaldeen Deeb, Alhareth M. Amro, Yousef Abu Asbeh

**Affiliations:** ^1^Faculty of Medicine, Al-Quds University, Jerusalem, Palestine; ^2^Thoracic Surgery Unit, Al-Ahli Hospital, Hebron, Palestine

**Keywords:** bronchoscopy, remimazolam, midazolam, sedation, systematic review, meta-analysis

## Abstract

**Background:**

Effective sedation is critical for bronchoscopy, ensuring patient comfort and procedural success. Midazolam, though widely used, has limitations such as longer induction and recovery times. Remimazolam, a novel ultra-short-acting benzodiazepine, offers rapid onset, faster recovery, and a safer profile, making it a potential alternative.

**Study design and methods:**

A systematic review and meta-analysis of four randomized controlled trials (RCTs) involving 630 patients compared remimazolam with midazolam for bronchoscopy sedation. Primary outcomes included induction time, recovery time, and rescue sedation rates. Secondary outcomes assessed procedural duration and adverse events. Random-effects models were used for analysis, and evidence quality was graded using GRADE criteria.

**Results:**

Remimazolam reduced induction time by 3.2 min (95% CI: −5.51 to −0.91, *p* = 0.006) and recovery time (SMD: -0.976, 95% CI: −1.48 to −0.47, *p* < 0.001) compared to midazolam. Patients receiving remimazolam required less rescue sedation (OR: 0.223, 95% CI: 0.107 to 0.467, *p* < 0.001). No significant differences were observed in bronchoscopy duration or the incidence of intraoperative and postoperative complications, including hypoxia, hypotension, and nausea.

**Conclusion:**

Remimazolam may reduce induction and recovery times and decrease the need for rescue sedation compared to midazolam. However, due to high heterogeneity and the limited number of studies, these findings should be interpreted with caution. Due to the limited number of studies and observed heterogeneity, further high-quality trials are necessary to confirm these findings.

**Clinical trial registration:**

Clinicaltrial.gov, identifier: CRD42024623846.

## Introduction

Bronchoscopy is a commonly used diagnostic and therapeutic procedure that frequently necessitates sufficient anesthesia to guarantee patient comfort, compliance, and procedural success (1). Agents like midazolam (the most frequently used) and propofol have historically been used for this purpose; nevertheless, their use is linked to drawbacks such as hemodynamic instability, respiratory depression, and delayed recovery. Remimazolam, a new benzodiazepine, has become a viable substitute for sedation during procedures, particularly bronchoscopies, in recent years (2).

Remimazolam is a short-acting benzodiazepine that has sedative, amnestic, and anxiolytic effects by binding to gamma-aminobutyric acid (GABA) receptors (3). Tissue esterases quickly break it down into an inactive metabolite, which results in a predictable and advantageous pharmacokinetic profile with little accumulation and a brief half-life that is context-sensitive (4, 5). It is appropriate for short interventions and high-turnover clinical environments because of these characteristics, which also help with its quick start and recovery.

Many recent studies have assessed the efficacy of remimazolam across various procedural settings, including gastrointestinal endoscopy, bronchoscopy, heart surgery, and pediatric anesthesia. According to these trials, it has better recovery durations and a lower risk of cardiovascular and respiratory side effects than traditional sedatives (6–8). Remimazolam may be especially helpful for individuals who are at risk of cardiovascular compromise because it has shown good hemodynamic stability in both adult and pediatric populations. Additionally, it has been shown to be beneficial in lowering surgical delirium and preoperative anxiety in pediatric patients (8).

Nonetheless, limitations have also been documented. Depending on patient-specific characteristics, such as cardiac disease and comorbidities, some studies found varying impacts on hemodynamics. Remimazolam has occasionally been linked to hypotension or under-sedation at larger dosages (9). Furthermore, the overall level of evidence remains low in some clinical domains, underscoring the need for further high-quality research.

Given the increasing evidence and expanding clinical application of remimazolam in different procedural contexts, there is a necessity to compile the available information regarding its safety and effectiveness. Additionally, randomized controlled trials assessing remimazolam for bronchoscopy have shown inconsistent results. These discrepancies highlight the necessity for a thorough synthesis of the current evidence to guide clinical decision-making. This systematic review and meta-analysis intend to offer a detailed assessment of remimazolam’s efficacy during bronchoscopy, in comparison to midazolam, concentrating on sedation quality, recovery profile, and occurrence of adverse events.

## Methodology

### Search strategy

This systematic review was registered on PROSPERO. The meta-analysis was conducted in accordance with the Preferred Reporting Items for Systematic Reviews and Meta-Analyses (PRISMA) 2020 guidelines (Supplementary Table S1) (10). The inclusion criteria comprised randomized controlled trials (RCTs) that compared remimazolam with midazolam for sedation in patients undergoing bronchoscopy, with no restrictions on language, country, gender, or age. Non-English studies were included if translated versions were available or could be produced through automated tools. Exclusion criteria encompassed non-human investigations (e.g., animal or *in vitro* studies), non-original research (such as systematic reviews or meta-analyses), various publication types (including theses, book chapters, editorials, conference papers, letters, and patents), as well as studies without extractable data or those that were duplicates.

A comprehensive search strategy was independently conducted by two authors (SD and AA) across multiple databases, namely MEDLINE (via PubMed), EMBASE (via Elsevier), the Cochrane Central Register of Controlled Trials (CENTRAL), Web of Science (WOS), Scopus, and Google Scholar. Details of this search strategy are provided in the supplementary file (Supplementary Table S2). The searches were conducted from November 16, 2024, to November 24, 2024. In addition, the reference lists of all eligible studies and relevant review articles were manually screened for additional citations. Conference proceedings and meeting abstracts were explored through Web of Science, EMBASE, and Google Scholar. Searches were conducted from the inception of each database until November 24, 2024, without any date or language limitations to ensure thorough coverage of pertinent literature.

References from each database were transferred to the EndNote reference manager, and duplicates were removed. Two authors independently reviewed the titles and abstracts against the predefined criteria. Potentially relevant articles were then assessed in full by two authors also. Any disagreements during this process were resolved by a third reviewer. Data extraction from the selected articles and supplementary materials was independently conducted by two authors (SD and AA) using a pretested standardized data extraction form designed to ensure consistency and completeness. Any disagreements in study selection or quality assessment were resolved by consultation with a third reviewer (YA). Finally, the reference lists of other reviews were checked to identify any additional studies that might meet the eligibility criteria.

### Data extraction

Data extraction for the meta-analysis comparing remimazolam and midazolam in bronchoscopy procedures focused on critical categories to ensure thorough examination of the reviewed studies. Two independent reviewers (SD and AA) performed data extraction separately using a standardized form. Any discrepancies or disagreements between the two reviewers were resolved through discussion with a third reviewer to ensure accuracy and consensus (YA).

Firstly, patient demographics were meticulously recorded, capturing essential information such as sample size, sex, age, and Body Mass Index (BMI). Procedural details were also vital, documenting key aspects such as the type and duration of the bronchoscopy procedure, along with instances of rescue sedative medication administration. The extraction included specific characteristics of sedation, detailing how remimazolam and midazolam were administered, including dosing protocols, induction, maintenance, recovery timings, and any additional doses given during the procedure. Additionally, the extraction included the American Society of Anesthesiologists (ASA) score to classify patient physical status.

Outcomes were a primary focus, with measures such as induction time, bronchoscopy duration, recovery time, and administration of rescue sedative medication being highlighted. Also highlighted as primary outcomes were hypoxia and hypotension. Secondary outcomes included tachycardia, hypertension, cough, nausea, and vomiting, providing insight into the safety profiles of the medications. Statistical analyses were also extracted, detailing mean and median doses of sedatives and timeframes for various procedural aspects, as well as a number of events for dichotomous outcomes. If certain data of interest is not mentioned in the article, we will contact the corresponding author to obtain further details or clarification. Each included study was assigned a unique article ID, with key details such as title, authors, and year of publication documented for reference.

### Quality assessment

The methodological quality of the included RCTs was evaluated using the Cochrane Collaboration’s Risk of Bias Tool (RoB2) (11). Two authors (SD and AA) performed this assessment independently, with any disagreements resolved by third author (YA). Each bias domain received a rating of low risk, high risk, or some concerns. Subsequently, the RobVis tool (12) was used to generate a visual summary of the risk of bias across the studies, presented in both a traffic light plot and a summary plot. A *p*-value of 0.05 was used to determine statistical significant.

### Statistical analysis

For dichotomous outcomes, the odds ratio (OR) and corresponding 95% confidence interval (CI) were calculated. Continuous outcomes were assessed using the mean difference (MD) or standard mean difference (SMD), both presented with 95% CIs. Heterogeneity was evaluated using the Cochran Q test and I^2^ statistics; *p* values below 0.1 and I^2^ values exceeding 50% were interpreted as indicators of significant heterogeneity. A *p*-value of 0.05 was used to determine statistical significance for the outcome analyses. When data were reported as a median with an interquartile range, they were converted into mean and standard deviation according to the formula proposed by Abbas et al. (13). A random-effects meta-analysis was conducted using Comprehensive Meta-Analysis V3 (CMA). Each study with extractable data about any outcome will enter the outcome analysis. We do a meta-analysis for every outcome that has analytical data from two or more studies. Publication bias was assessed using funnel plots and Egger’s tests for outcomes that included at least three studies, with a 2-tailed *p*-value< 0.05 denoting significance. Furthermore, a sensitivity analysis was performed by excluding one study at a time to ensure that the overall findings were not driven by any single investigation.

### Certainty of evidence

The quality of evidence for each outcome was evaluated independently by two authors (SD and AA) using the Grading of Recommendations, Assessment, Development, and Evaluation (GRADE) tool (14). Any disagreements were resolved through discussions with third author (YA). Based on evaluations of bias of included study, indirectness, inconsistency, imprecision, and publication bias, the overall certainty of the evidence was rated high, moderate, low, or very low.

## Results

**Figure 1:**
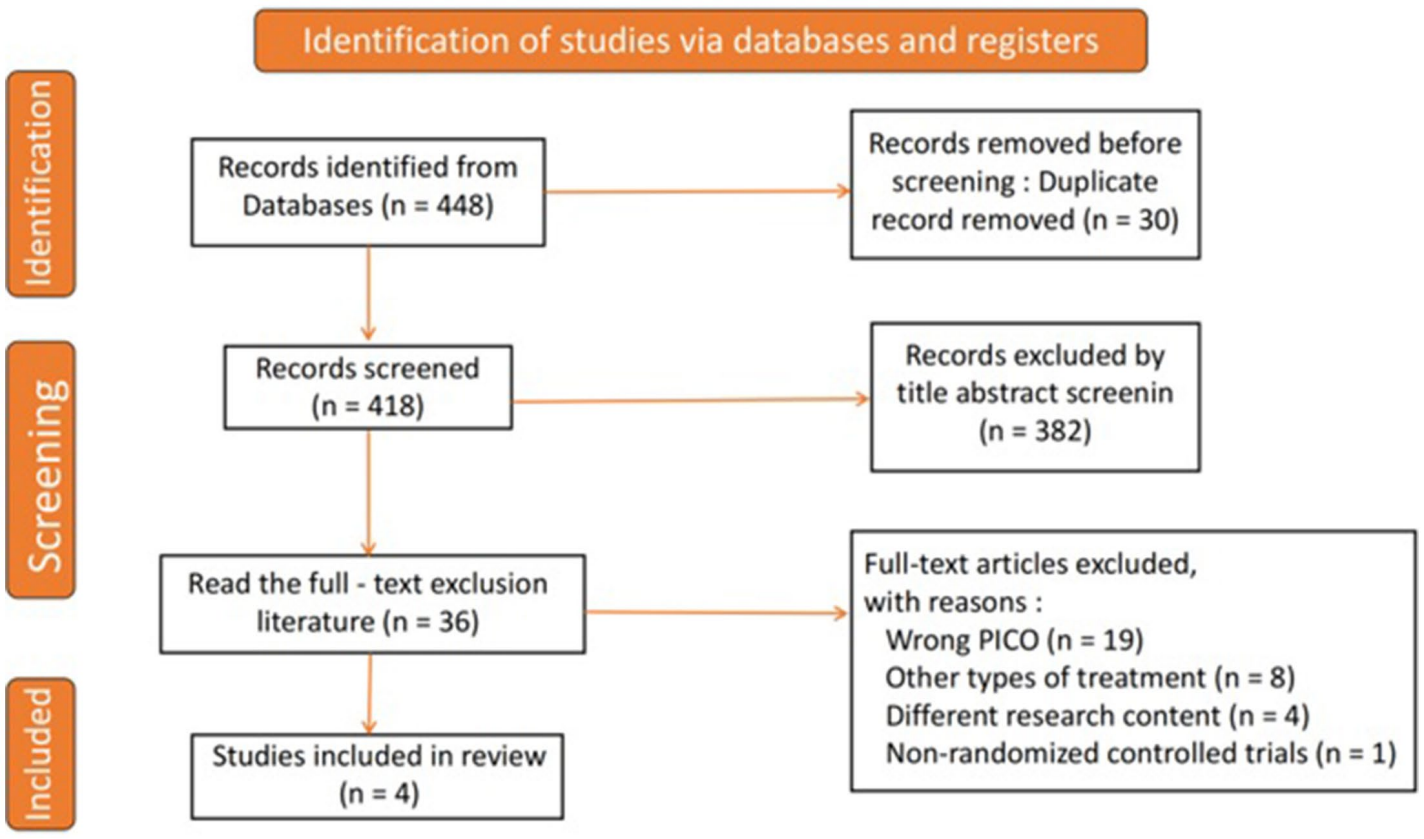
Flow diagram of study searching and selection process.

As illustrated in Figure 1, a comprehensive literature search yielded a total of 448 studies. After eliminating 30 duplicates, 418 unique studies were available for screening. From this group, 382 studies were excluded based on a review of titles and abstracts, resulting in 36 studies selected for full-text evaluation. After a detailed assessment according to the inclusion and exclusion criteria, 32 studies were discarded. Ultimately, four RCTs were included in the analysis (15–18).

Table 1 provides an overview of the characteristics of the four studies included in this meta-analysis. Two of the studies were conducted in China, one in South Korea, and one in the United States. The publication dates of these studies range from 2019 to 2024. A total of 630 patients were analyzed, with 52.38% of the participants being male. Of these, 432 patients were sedated with remimazolam and 198 with midazolam. Also, in the supplementary file (Supplementary Table S3), the table represents the summary of adverse effects with the number of patients for them in each study separately.

**Table 1:** Basic characteristics of included studies.

Ref	Country	ASA status	Total No	Remimazolam group					Midazolam group				
				No	Age mean (Y)	(n) M/F	BMI	Dose	No	Age mean (Y)	(n) M/F	BMI	Dose
Pastis et al. (15)	United State	I-III	372	303	62.7 ± 12.09	139/164	28.4 ± 6.39	Initial dose: 5 mg and fentanyl 25–75 μg; Top-up dose: 2.5 mg midazolam for rescue	69	61.5 ± 14.03	35/34	28 ± 5.79	Initial dose: 1–1.75 mg and fentanyl 25–75 μg; Top-up dose: 0.5–1 mg midazolam for rescue
Kim et al. (16)	South Korea	I-III	100	49	65 ± 14.07	31/ 18	23.47 ± 3.44	A < 60 y or W > 50 Kg: 5 mg, A ≥ 60 y or W < 50 Kg: 3 mg; Top-up dose: 2.5 mg	51	68 ± 11.11	30/21	21.9 ± 3.2	A < 60 y or W > 50 Kg: 3 mg, A ≥ 60 y or W < 50 Kg: 2 mg; Top-up dose: 0.5 mg
Huang et al. (17)	China	I-II	64	34	54.65 ± 13.23	22/ 12	22.26 ± 3.65	Initial dose: 0.2 mg/kg and fentanyl 0.5 μg/kg; Top-up dose: 25 μg fentanyl	30	57.37 ± 12.36	18/12	23.2 ± 3.73	Initial dose: 0.075 mg/kg and fentanyl 0.5 μg/kg; Top-up dose: 25 μg fentanyl
Wu et al. (18)	China	I-III	94	46	70.37 ± 4.07	26/20	22.09 ± 3.65	Initial dose: 0.135 mg/kg and alfentanyl 18 μg/kg; pfopofol for rescue	48	69.21 ± 3.59	29/19	22.19 ± 3.06	Initial dose: 0.045 mg/kg and alfentanyl 18 μg/kg; pfopofol for rescue

N, number; ASA, American Society of Anesthesiologists; Y, years; M, male; F, female.

**Figure 2:**
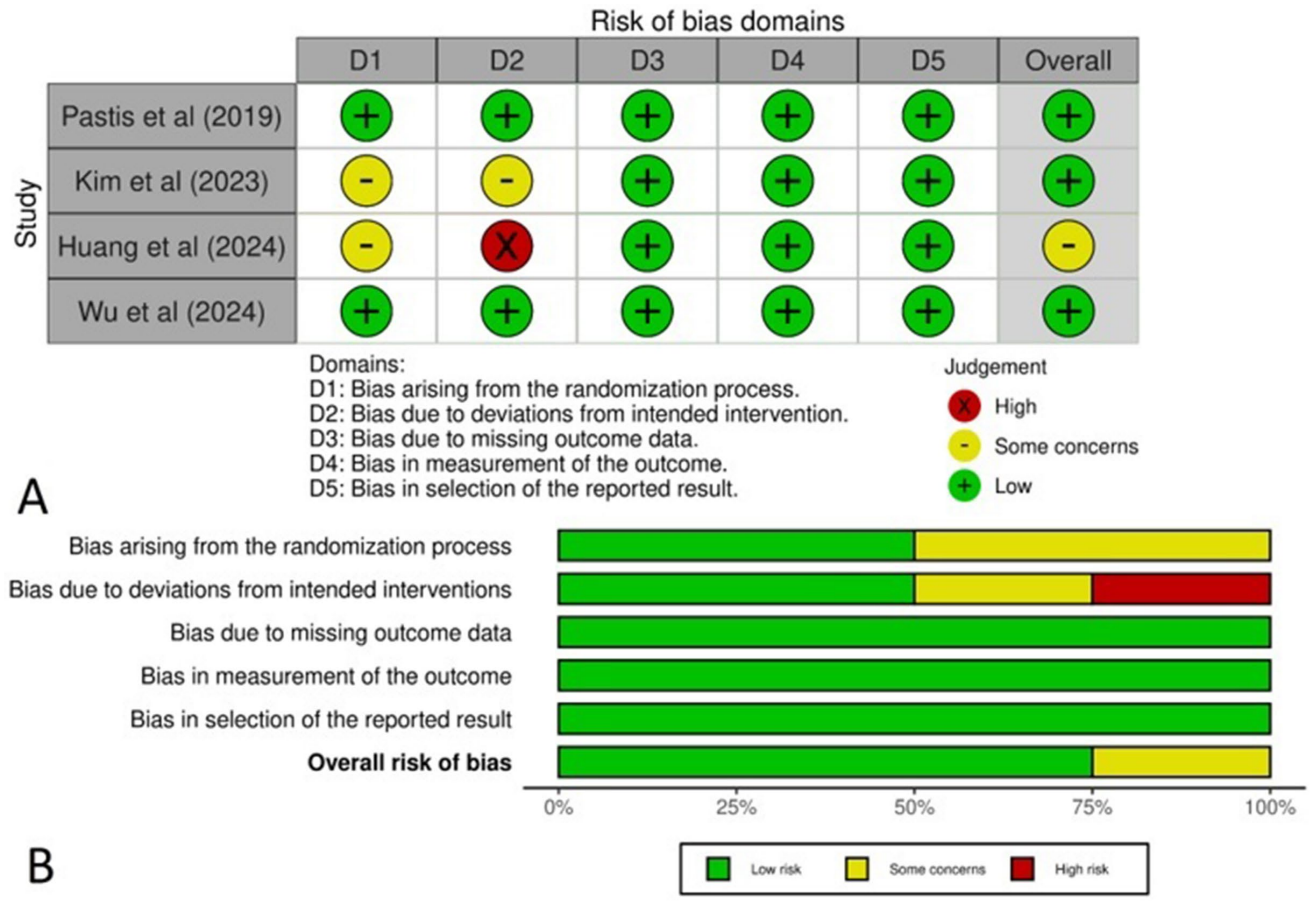
**(A)** Traffic light plot representing the risk of bias across the studies, **(B)** Summary plot representing the total risk of bias in each domain.

The Cochrane method was applied to assess the risk of bias in the RCTs, with the findings represented in a traffic light plot (Figure 2A) and a summary plot (Figure 2B). All four studies demonstrated a low risk of bias, with the exception of the Huang et al. study, which was categorized as having “some concerns.” As the randomization process (50%), intervention assignment (50%) and missing outcome data (100%). Additionally, 100% of the studies had a low risk of bias in outcome measurement, while 100% were rated as low risk for selecting reported results.

### Results of the meta-analysis

In four studies, a total of 630 patients were enrolled to compare the safety and efficacy of remimazolam and midazolam. The analysis encompassed both primary and secondary outcomes, including procedure times, the need for rescue sedatives, and the incidence of adverse events. Table 2 provides a summary of the meta-analysis results for all included outcomes.

**Table 2:** The summary of meta-analysis results for the comparison between Remimazolam and Midazolam for all out comes.

Outcome	No. of patients	Effect size measure	Effect size	95% CI	*p*-value	Heterogeneity (I^2^)	*Q* value	*p*-value (Heterogeneity)
Induction time (Min)	630	MD	−3.214	(−5.513 to −0.914)	0.006	97.666	128.523	< 0.001
Bronchoscopic duration (Min)	630	MD	0.270	(−0.674 to 1.214)	0.575	21.868	3.84	0.279
Recovery time (Min)	536	SMD	−0.976	(−1.483 to −0.469)	< 0.001	80.118	10.06	0.007
Rescue Sedative use	466	OR	0.223	(0.107 to 0.467)	< 0.001	54.405	2.193	0.139
Intraoperative hypoxia	630	OR	1.286	(0.745 to 2.219)	0.367	< 0.001	0.786	0.853
Intraoperative hypotension	473	OR	0.751	(0.449 to 1.258)	0.277	< 0.001	0.054	0.816
Intraoperative hypertension	436	OR	0.701	(0.235 to 2.088)	0.523	65.143	2.869	0.09
Intraoperative tachycardia	536	OR	0.916	(0.233 to 3.608)	0.901	< 0.001	0.698	0.706
Intraoperative cough	158	OR	0.787	(0.410 to 1.511)	0.472	< 0.001	0.155	0.693
Postoperative nausea	466	OR	1.468	(0.455 to 4.736)	0.52	< 0.001	0.015	0.902
Postoperative vomiting	466	OR	0.926	(0.217 to 3.944)	0.917	< 0.001	0.71	0.399

OR, odds ratio; Min, Minutes; MD, Mean difference; SMD, standardized mean difference; No, number.

**Figure 3:**
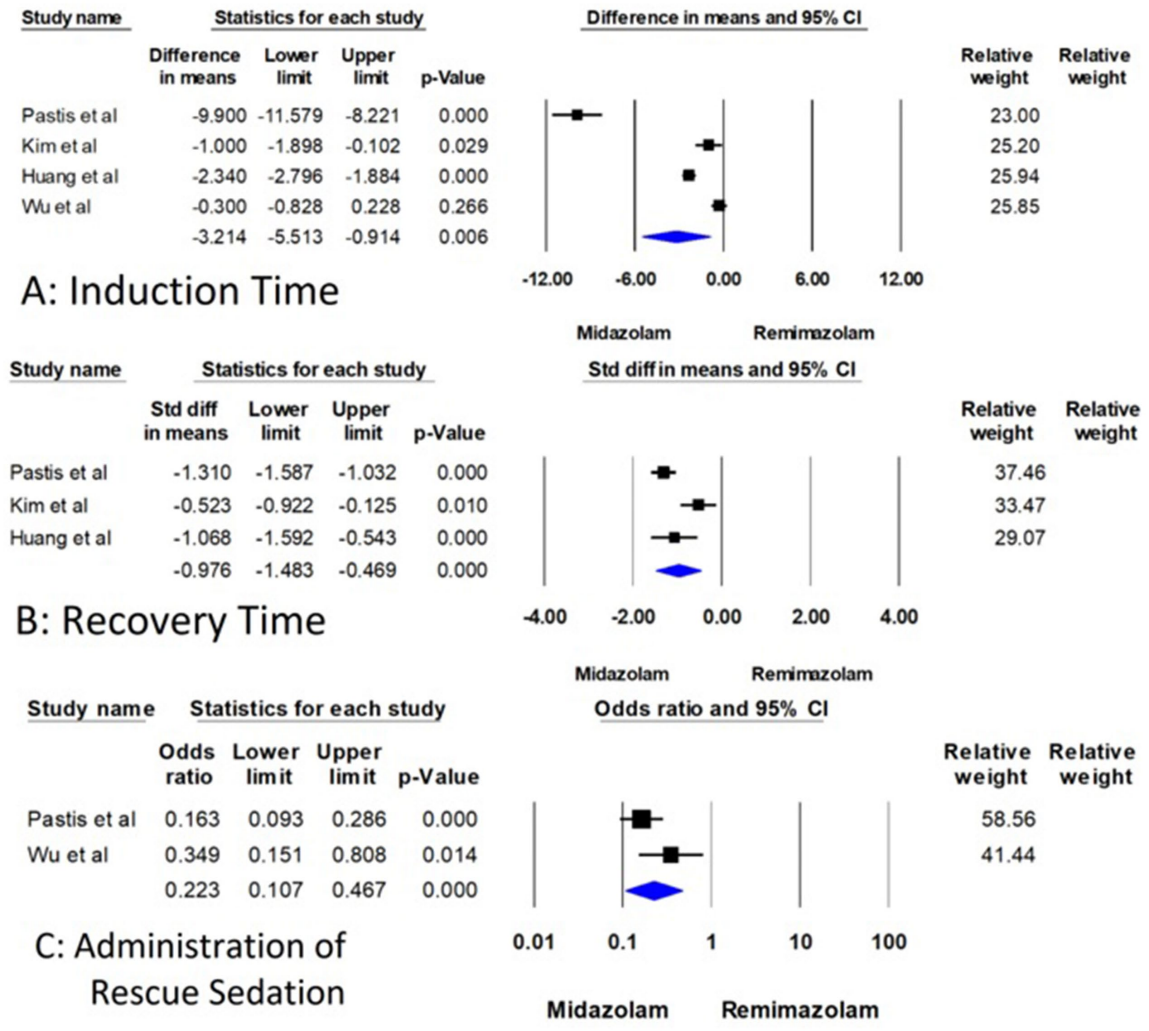
Forest plot for comparison of induction time, recovery time, and administration of rescue sedation between remimazolam and midazolam.

For induction time, the four studies with 630 patients compared remimazolam to midazolam (15–18). The mean difference (MD) in induction time was found to be −3.214 min. This result was statistically significant with a 95% confidence interval (CI) of (−5.513 to −0.914) and a *p*-value of 0.006, which indicates that remimazolam reduces the induction time by approximately 3.2 min compared to midazolam (Figure 3A). However, the analysis exhibited high heterogeneity with an I^2^-value of 97.666 and a Q-value of 128.523, with a p-value for heterogeneity less than 0.001. However, substantial heterogeneity was present (I^2^ = 97.7%), suggesting considerable variation across studies. This reduces the reliability of the pooled estimate and warrants cautious interpretation.

In terms of bronchoscopy duration, the comparison was evaluated across the four studies involving a total of 630 patients (15–18). The mean difference (MD) was 0.270 min, with 95% CI (−0.674 to 1.214) and *p*-value = 0.575, indicating no statistical significance (Supplementary Figure S1). Heterogeneity for this outcome was low (I^2^ = 21.9%, Q = 3.840, *p* = 0.279).

Three studies involving 536 patients examined recovery time, but the measurement points for recovery time varied across the studies (15–17). Therefore, the standardized mean difference (SMD) was used for this analysis to account for these differences. The SMD for recovery time was −0.976, 95% CI (−1.483 to −0.469), and a highly significant *p*-value of less than 0.001 (Figure 3B). This indicates a substantial decrease in recovery time when using remimazolam compared to midazolam, although the analysis demonstrated high heterogeneity (I^2^ = 80.118, Q = 10.060, *p* = 0.007). Although the effect size was statistically significant, the analysis also showed high heterogeneity (I^2^ = 80.1%), which may reflect differences in measurement time points, patient populations, or dosing strategies across trials.

Regarding the administration of rescue sedatives, only two RCTs discuss the rescue sedatives with 536 patients (15, 18). The odds ratio (OR) for the use of rescue sedative medication was 0.223, 95% CI (0.107 to 0.467), and a significant *p*-value of less than 0.001 (Figure 3C). These findings indicate that remimazolam significantly reduces the likelihood of requiring additional sedative medication during the procedure. The heterogeneity for this outcome was not statistically significant (I^2^ = 54.405, Q = 2.193, *p* = 0.139) (Figure 3).

Intraoperative complications, including hypoxia, hypotension, hypertension, tachycardia, and cough, as well as postoperative side effects, including nausea and vomiting, were found to be non-significant, with *p*-values exceeding 0.05 (Supplementary Figure S2-S8). This indicates that there is no significant difference between remimazolam and midazolam in terms of intraoperative complications and postoperative side effects. As for heterogeneity, it was very low for all these outcomes except for hypertension, which showed moderate heterogeneity (details of the analysis for each outcome are provided in Table 2).

To assess the publication bias and Egger test results, funnel plots and Egger test results related to each outcome are found in the supplementary file (Supplementary Figures S9–S13). Egger’s test was not statistically significant in induction time, bronchoscopy duration, recovery time, hypoxia, and tachycardia (2-tailed *p*-value > 0.05).

In the sensitivity analysis, there were no significant changes in the results for any outcome after excluding individual studies, as shown in the forest plots in the supplementary file (Supplementary Figures S14–S24), except for induction time. When the studies by Pastis et al. or Huang et al. were removed, the difference in induction time between remimazolam and midazolam was not significant (15, 17). However, excluding the studies by Kim et al. or Wu et al. resulted in a mean difference (MD) of −4.010 (95% CI = −7.056 to −0.964, *p* = 0.010) and −4.297 (95% CI = −7.680 to −0.914, *p* = 0.013), respectively (15, 17).

### Certainty of evidence

For certainty of evidence, the risk of bias was a significant concern, with all outcomes being downgraded due to the inclusion of studies at high risk of bias, which could affect the interpretation of results. Additionally, inconsistency was noted in two outcomes, as indicated by considerable heterogeneity (I^2^ > 50%). For outcomes assessing rare events, the certainty was downgraded for imprecision due to insufficient total sample size and wide 95% CIs, limiting the reliability of the estimates. Overall, the evidence was rated as high certainty for five outcomes, moderate certainty for four outcomes, and low certainty for two outcomes, reflecting the impact of these limitations on the overall assessment (Table 3).

**Table 3:** Summary of outcomes and certainty of evidence based on the Grading of recommendations assessment, development and evaluation (GRADE) approach.

Outcome	Number of Studies	Risk of bias	Inconsistency	Indirectness	Imprecision	No of patients			
						Remimazolam	Midazilam	Effect estimate (95% CI)	Grade
Induction time	4 (15–18)	Low	Serious (I^2^ = 97.666),b	Not Serious	Serious	432	198	−3.214 min (−5.513 to −0.914)	Low ⨁ ⨁ ◯ ◯
Bronchoscopic duration	4 (15–18)	Low	Not serious	Not serious	Not serious	432	198	0.270 min (−0.674 to 1.214)	High ⨁ ⨁ ⨁ ⨁
Recovery time	3 (15–17)	Low	Moderate (I^2^ = 80.118),b	Not serious	Not serious	129	129	−0.976 (−1.483 to −0.469)	High ⨁ ⨁ ⨁ ⨁
Rescue sedative use	2 (15, 18)	Low	Moderate (I^2^ = 54.405),b	Not serious	Not serious	64/349	66/117	0.223 (0.107 to 0.467)	High ⨁ ⨁ ⨁ ⨁
Hypoxia	4 (15–18)	Low	Not serious	Not serious	Not serious	78/432	21/198	1.286 (0.745 to 2.219)	High ⨁ ⨁ ⨁ ⨁
Hypotension	2 (15, 16)	Low	Not serious	Not serious	Not serious	128/352	35/120	0.751 (0.449 to 1.258)	High ⨁ ⨁ ⨁ ⨁
Hypertension	2 (15, 17)	Low	Moderate (I^2^ = 65.143),b	Not Serious	Serious	191/337	51/99	0.701 (0.235 to 2.088)	Moderate ⨁ ⨁ ⨁ ◯
Tachycardia	3 (15–17)	Low	Not serious	Not serious	Serious	7/386	4/150	0.916 (0.233 to 3.608)	Moderate ⨁ ⨁ ⨁ ◯
Cough	2 (17, 18)	Some concern	Not serious	Not serious	Serious	43/80	47/78	0.787 (0.410 to 1.511)	Low ⨁ ⨁ ◯ ◯
Postoperative nausea	2 (15, 18)	Low	Not serious	Not serious	Serious	15/349	4/117	1.468 (0.455 to 4.736)	Moderate ⨁ ⨁ ⨁ ◯
Postoperative vomiting	2 (15, 18)	Low	Not serious	Not serious	Serious	7/398	2/168	0.926 (0.217 to 3.944)	Moderate ⨁ ⨁ ⨁ ◯

CI, confidence interval. a. The proportion of information from studies at high risk of bias is sufficient to affect the interpretation of results, b. I suggested considerable heterogeneity, c. For comparison of the incidence of rare events, the total sample size appeared insufficient, and the 95% Cl was too wide.

## Discussion

This systematic review and meta-analysis evaluated the efficacy and safety of remimazolam compared to midazolam for sedation during bronchoscopy, synthesizing data from four randomized controlled trials involving 630 patients (432 received remimazolam and 198 received midazolam). The findings provide compelling evidence supporting remimazolam as a promising alternative sedative, particularly in procedural and diagnostic contexts. Notably, remimazolam significantly reduced induction time (MD = −3.214 min, 95% CI: −5.513 to −0.914, *p* = 0.006) and recovery time (SMD = −0.976, 95% CI: −1.483 to −0.469, *p* < 0.001), both of which are key metrics in optimizing procedural workflow, minimizing sedation-related delays, and improving patient throughput. Furthermore, patients receiving remimazolam had a significantly lower likelihood of requiring rescue sedation (OR = 0.223, 95% CI: 0.107 to 0.467, p < 0.001), indicating enhanced sedation stability. Despite these improvements in workflow, the choice of sedative did not significantly affect bronchoscopy duration (MD = 0.270 min, *p* = 0.575), reinforcing the procedural equivalence of remimazolam and midazolam in terms of procedural length.

While the results suggest that remimazolam may offer advantages in terms of induction time, recovery time, and the need for rescue sedation, the reliability of these findings is limited by the considerable heterogeneity observed in the meta-analyses. For example, both induction and recovery times exhibited high I^2^ values, indicating significant between-study variability. This heterogeneity may result from differences in study design, remimazolam dosing strategies, co-sedation protocols (e.g., use of fentanyl or alfentanil), and varying definitions of outcomes across studies. As such, although the pooled estimates were statistically significant, the inconsistency across trials reduces the certainty of the evidence and calls for cautious interpretation. The limited number of included RCTs also constrains our ability to perform subgroup analyses or meta-regression to explore potential sources of heterogeneity.

In terms of safety, remimazolam demonstrated a comparable adverse event profile to midazolam. No statistically significant differences were observed in intraoperative complications such as hypoxia (OR = 1.286, *p* = 0.367), hypotension (OR = 0.751, *p* = 0.277), hypertension, tachycardia, or intraoperative cough, all of which exhibited non-significant effect sizes and minimal heterogeneity. Postoperative events, including nausea (OR = 1.468, *p* = 0.520) and vomiting (OR = 0.926, *p* = 0.917), were also similar between the two groups. These findings reinforce remimazolam’s clinical safety and tolerability in procedural sedation settings.

Although one prior meta-analysis assessed the efficacy and safety of remimazolam in bronchoscopy, it combined various comparators—such as propofol, dexmedetomidine, and midazolam—under the umbrella of conventional sedatives. It included only a single study that directly compared remimazolam with midazolam, limiting the applicability of its findings to this specific comparison. In contrast, our study is the first to provide a dedicated meta-analysis of remimazolam versus midazolam in bronchoscopic sedation, offering more focused and clinically relevant evidence to guide sedation protocols (19).

Our findings align with existing literature. For example, in a phase IIb study of patients undergoing colonoscopy, remimazolam was associated with faster induction time than midazolam (20). Similarly, another systematic review and meta-analysis demonstrated that remimazolam not only shortened recovery time but also significantly reduced the need for rescue sedation in gastrointestinal endoscopic procedures, mirroring our results (21). Kim et al. further found that patients undergoing non-biopsy bronchoscopic procedures reported greater satisfaction when sedated with remimazolam compared to midazolam (16). These advantages are particularly beneficial in outpatient settings, where shorter procedural times translate into improved efficiency, faster discharge, and better resource utilization. By reducing the need for additional sedation and promoting quicker recovery, remimazolam contributes to a smoother procedural experience and potentially enhances patient satisfaction.

While our analysis did not show significant differences in intraoperative complications, other studies have found some safety advantages. For instance, a meta-analysis comparing remimazolam with midazolam in gastrointestinal endoscopy reported a reduction in adverse events, including hypotension, hypertension, bradycardia, tachycardia, and hypoxia, favoring remimazolam (21). Furthermore, remimazolam has been shown to be superior to propofol in terms of reducing the incidence of hypotension and respiratory depression during both bronchoscopy and general anesthesia procedures (19, 22). These results suggest that remimazolam may offer a broader safety margin in specific clinical contexts, especially in patients at higher risk for hemodynamic or respiratory instability.

Additionally, both remimazolam and midazolam share flumazenil as a specific antagonist, allowing for the rapid reversal of prolonged sedation or adverse reactions, when necessary, which further enhances the safety margin of remimazolam in clinical practice (4). Kim et al. also reported no significant difference in the need for flumazenil between the two drugs during bronchoscopy procedures (16).

Regarding postoperative side effects, our meta-analysis revealed no significant differences in the incidence of nausea and vomiting between the two sedatives. However, Pastis et al. found that remimazolam was associated with superior neuropsychiatric recovery five minutes after patients regained full alertness compared to midazolam (15). Despite this early cognitive advantage, there were no statistically significant differences in postoperative recall or patients’ willingness to undergo reexamination, as reported by another trial (18). These findings suggest that remimazolam is safe and well-tolerated not only intraoperatively but also in the postoperative period, with a comparable—and potentially more favorable—safety profile relative to midazolam.

Among elderly patients, a particularly vulnerable population, remimazolam demonstrated additional clinical benefits. Wu et al. reported that remimazolam significantly outperformed midazolam in achieving deep sedation during diagnostic flexible bronchoscopy, with higher sedation success and a lower incidence of postoperative oversedation (18). The faster onset and shorter recovery associated with remimazolam are critical advantages in this group, reducing the risk of prolonged sedation and associated complications. Moreover, a separate study comparing remimazolam and propofol emphasized the favorable safety profile of remimazolam in elderly patients, although careful monitoring and individualized dosing remain essential due to the pharmacodynamic variability in this age group (23).

Moreover, from a financial perspective, these results suggest that remimazolam reduces the cost of bronchoscopy sedation. This aligns with findings from a Danish study comparing remimazolam and midazolam in bronchoscopy sedation, which demonstrated a 22% reduction in the cost of successful bronchoscopy sedation, including the cost of the drug itself (24). The cost-effectiveness is largely attributable to the significantly shorter induction and recovery times, which reduce procedural downtime and increase turnover efficiency. These results underscore the pharmacoeconomic value of remimazolam in high-throughput settings.

The optimal sedative for bronchoscopy should ideally combine rapid onset, ease of administration, short procedural duration, predictable recovery, and a well-characterized safety profile (25). While midazolam has historically met many of these criteria, it is limited by prolonged sedation and extended recovery, which can delay discharge and increase resource utilization. Our findings suggest that remimazolam overcomes these limitations, providing an adjustable depth and duration of sedation while maintaining safety comparable to midazolam. Its efficiency, patient-friendly profile, and cost-effectiveness support its prioritization as a first-line agent for bronchoscopy sedation.

Despite these encouraging findings, several limitations must be acknowledged. First, some outcomes displayed substantial heterogeneity, likely due to variability in dosing strategies and co-administration of opioids across studies. Furthermore, the studies included in this analysis did not follow standardized criteria for defining induction and recovery times. In several trials, the depth of sedation at induction was not clearly stated, and recovery timing may have been assessed at different sedation levels. The inconsistent use of reversal agents such as flumazenil could have further affected recovery time assessments, reducing comparability across studies. It should also be noted that one of the included studies was a preprint that had not undergone peer review at the time of analysis. Although the inclusion of preprints can help reduce publication bias and reflect the most current data, their methodological quality is not yet formally verified. This may limit the overall strength and certainty of our conclusions. Second, our analysis included only four randomized controlled trials, and most were conducted in specific geographic regions, potentially limiting the generalizability of the results to broader populations. Third, the short follow-up durations in the included studies restrict the assessment of long-term adverse events and safety outcomes. Finally, important patient-centered outcomes such as satisfaction, cognitive recovery, and willingness to undergo repeat procedures were not uniformly reported. These limitations underscore the need for future multicenter RCTs with standardized protocols, broader population diversity, and longer follow-up to fully establish the clinical and economic utility of remimazolam in bronchoscopy sedation.

The findings of this meta-analysis highlight the clinical relevance of remimazolam as a safe and effective alternative to midazolam for sedation during bronchoscopy, particularly in outpatient and high-throughput settings. Its favorable profile—characterized by faster induction, shorter recovery, and a reduced need for rescue sedation—supports its potential to streamline procedural workflows and enhance patient safety and satisfaction. These advantages are particularly meaningful for vulnerable populations such as the elderly and those with cardiopulmonary comorbidities.

However, it is important to interpret these results with caution. The analysis included only four randomized controlled trials, and some outcomes such as induction and recovery times exhibited substantial heterogeneity. This variability may reflect differences in sedation protocols, remimazolam dosing regimens, and concomitant opioid use across studies. Consequently, while our results are promising, they cannot be considered definitive. Additionally, differences in the type and dose of opioids co-administered during bronchoscopy, such as fentanyl and alfentanil, likely contributed to clinical heterogeneity. These variations in adjunctive medication protocols may have influenced sedation depth, recovery time, and the overall effect size observed in our analysis.

We therefore strongly recommend the design and implementation of large-scale, rigorously controlled randomized trials to further evaluate remimazolam’s efficacy and safety, with particular attention to dose standardization, opioid co-administration, and comprehensive reporting of patient-centered outcomes. Such studies are essential to validate our findings and support evidence-based updates to bronchoscopy sedation guidelines.

If confirmed in broader clinical settings with standardized protocols, remimazolam could represent a breakthrough in bronchoscopy by accelerating both diagnostic and therapeutic processes, ultimately transforming procedural sedation into a safer and more efficient practice.

## Conclusion

This systematic review and meta-analysis of four RCTs (630 patients) demonstrates that remimazolam significantly reduces induction and recovery times versus midazolam, without prolonging bronchoscopy duration or increasing intra- or postoperative complications. It also lowers the need for rescue sedation, underscoring its potential to streamline procedural workflows. Nevertheless, the high heterogeneity observed in some outcomes—attributable to varying dosing regimens and opioid use—underscores the need for well-designed randomized trials to establish optimal dosing strategies and standardized protocols. Remimazolam shows potential as an alternative to midazolam in outpatient bronchoscopy, with potential benefits for patient turnover, safety, and cost-effectiveness. Future studies should broaden patient populations, harmonize sedation regimens, and evaluate long-term outcomes to confirm and extend these findings. Given the limited number of studies and observed heterogeneity, further large-scale randomized trials are warranted to confirm these findings and guide clinical recommendations.

## Data Availability

The original contributions presented in the study are included in the article/Supplementary material, further inquiries can be directed to the corresponding author/s.
