# Supplementary material for: The comparison of remimazolam and midazolam in bronchoscopic sedation: a systematic review and meta-analysis of randomized controlled trials
Source: Front Med (Lausanne). 2025 Jul 8;12:1581280. doi: 10.3389/fmed.2025.1581280 (PMC12279844; doi:10.3389/fmed.2025.1581280)
Supplement: Supplementary file 1 [file Table_1.docx]

**Index**

PRISMA-2020 Checklist**:** Table S1

Search Strategy: Table S2

Studies and Participant’s Characteristics: Table S3

Results of the meta-analysis: Figure S1-S8

Publication Bias: Figure S9-S13

Sensitivity analysis: Figure S14-S24

**PRISMA-2020 Checklist**

**Table S1: PRISMA-2020**

| **Section and Topic** | **Item #** | **Cshecklist item** | **Location where item is reported** |
| --- | --- | --- | --- |
| **TITLE** | | |  |
| Title | 1 | Identify the report as a systematic review. | Page 1 |
| **ABSTRACT** | | |  |
| Abstract | 2 | See the PRISMA 2020 for Abstracts checklist. | Page 1 |
| **INTRODUCTION** | | |  |
| Rationale | 3 | Describe the rationale for the review in the context of existing knowledge. | Page 2 |
| Objectives | 4 | Provide an explicit statement of the objective(s) or question(s) the review addresses. | Page 2 |
| **METHODS** | | |  |
| Eligibility criteria | 5 | Specify the inclusion and exclusion criteria for the review and how studies were grouped for the syntheses. | Page 3 |
| Information sources | 6 | Specify all databases, registers, websites, organisations, reference lists and other sources searched or consulted to identify studies. Specify the date when each source was last searched or consulted. | Page 3 |
| Search strategy | 7 | Present the full search strategies for all databases, registers and websites, including any filters and limits used. | Supplementary Table S1 |
| Selection process | 8 | Specify the methods used to decide whether a study met the inclusion criteria of the review, including how many reviewers screened each record and each report retrieved, whether they worked independently, and if applicable, details of automation tools used in the process. | Page 3 |
| Data collection process | 9 | Specify the methods used to collect data from reports, including how many reviewers collected data from each report, whether they worked independently, any processes for obtaining or confirming data from study investigators, and if applicable, details of automation tools used in the process. | Page 4 |
| Data items | 10a | List and define all outcomes for which data were sought. Specify whether all results that were compatible with each outcome domain in each study were sought (e.g. for all measures, time points, analyses), and if not, the methods used to decide which results to collect. | Page 4 |
|  | 10b | List and define all other variables for which data were sought (e.g. participant and intervention characteristics, funding sources). Describe any assumptions made about any missing or unclear information. | Page 4 |
| Study risk of bias assessment | 11 | Specify the methods used to assess risk of bias in the included studies, including details of the tool(s) used, how many reviewers assessed each study and whether they worked independently, and if applicable, details of automation tools used in the process. | Page 4 |
| Effect measures | 12 | Specify for each outcome the effect measure(s) (e.g. risk ratio, mean difference) used in the synthesis or presentation of results. | Page 4 |
| Synthesis methods | 13a | Describe the processes used to decide which studies were eligible for each synthesis (e.g. tabulating the study intervention characteristics and comparing against the planned groups for each synthesis (item #5)). | Page 4 |
|  | 13b | Describe any methods required to prepare the data for presentation or synthesis, such as handling of missing summary statistics, or data conversions. | Page 4 |
|  | 13c | Describe any methods used to tabulate or visually display results of individual studies and syntheses. | Page 4 |
|  | 13d | Describe any methods used to synthesize results and provide a rationale for the choice(s). If meta-analysis was performed, describe the model(s), method(s) to identify the presence and extent of statistical heterogeneity, and software package(s) used. | Page 4 |
|  | 13e | Describe any methods used to explore possible causes of heterogeneity among study results (e.g. subgroup analysis, meta-regression). | Page 4 |
|  | 13f | Describe any sensitivity analyses conducted to assess robustness of the synthesized results. | Page 4 |
| Reporting bias assessment | 14 | Describe any methods used to assess risk of bias due to missing results in a synthesis (arising from reporting biases). | Page 4 |
| Certainty assessment | 15 | Describe any methods used to assess certainty (or confidence) in the body of evidence for an outcome. | Page 5 |
| **RESULTS** | | |  |
| Study selection | 16a | Describe the results of the search and selection process, from the number of records identified in the search to the number of studies included in the review, ideally using a flow diagram. | Figure 1 |
|  | 16b | Cite studies that might appear to meet the inclusion criteria, but which were excluded, and explain why they were excluded. | Figure 1 |
| Study characteristics | 17 | Cite each included study and present its characteristics. | Page 5,  Table 1 |
| Risk of bias in studies | 18 | Present assessments of risk of bias for each included study. | Figure 2 |
| Results of individual studies | 19 | For all outcomes, present, for each study: (a) summary statistics for each group (where appropriate) and (b) an effect estimate and its precision (e.g. confidence/credible interval), ideally using structured tables or plots. | Table 2,  Figure 3,  Supplementary Figures S1-S8 |
| Results of syntheses | 20a | For each synthesis, briefly summarise the characteristics and risk of bias among contributing studies. | Table 3 |
|  | 20b | Present results of all statistical syntheses conducted. If meta-analysis was done, present for each the summary estimate and its precision (e.g. confidence/credible interval) and measures of statistical heterogeneity. If comparing groups, describe the direction of the effect. | Page 6,7, Table 2,  Figure 3  Supplementary Figures S1-S8 |
|  | 20c | Present results of all investigations of possible causes of heterogeneity among study results. | Page 7, Supplementary Figure S14–S24 |
|  | 20d | Present results of all sensitivity analyses conducted to assess the robustness of the synthesized results. | Page 7, Supplementary Figure S14–S24 |
| Reporting biases | 21 | Present assessments of risk of bias due to missing results (arising from reporting biases) for each synthesis assessed. | Page 7, Supplementary Figure S9-S13 |
| Certainty of evidence | 22 | Present assessments of certainty (or confidence) in the body of evidence for each outcome assessed. | Table 3 |
| **DISCUSSION** | | |  |
| Discussion | 23a | Provide a general interpretation of the results in the context of other evidence. | Page 7,8 |
|  | 23b | Discuss any limitations of the evidence included in the review. | Page 9 |
|  | 23c | Discuss any limitations of the review processes used. | Page 9 |
|  | 23d | Discuss implications of the results for practice, policy, and future research. | Page 10 |
| **OTHER INFORMATION** | | |  |
| Registration and protocol | 24a | Provide registration information for the review, including register name and registration number, or state that the review was not registered. | Page 2 |
|  | 24b | Indicate where the review protocol can be accessed, or state that a protocol was not prepared. | Page 2 |
|  | 24c | Describe and explain any amendments to information provided at registration or in the protocol. | None |
| Support | 25 | Describe sources of financial or non-financial support for the review, and the role of the funders or sponsors in the review. | Page 10 |
| Competing interests | 26 | Declare any competing interests of review authors. | Page 11 |
| Availability of data, code and other materials | 27 | Report which of the following are publicly available and where they can be found: template data collection forms; data extracted from included studies; data used for all analyses; analytic code; any other materials used in the review. | upon request |

*From:*  Page MJ, McKenzie JE, Bossuyt PM, Boutron I, Hoffmann TC, Mulrow CD, et al. The PRISMA 2020 statement: an updated guideline for reporting systematic reviews. BMJ 2021;372:n71. doi: 10.1136/bmj.n71. This work is licensed under CC BY 4.0. To view a copy of this license, visit <https://creativecommons.org/licenses/by/4.0/>

**Search Strategy**

**Table S2. Search strategy until 24/11/2024**

| **Our search strategy in Pubmed: 71** | |
| --- | --- |
| (("remimazolam"[Supplementary Concept] OR "remimazolam"[All Fields] OR (("ONO"[All Fields] AND "2745"[All Fields]) OR "ONO2745"[All Fields] OR "ONO-2745"[All Fields] OR ("CNS"[All Fields] AND "7056"[All Fields]) OR ("methyl"[All Fields] AND "3"[All Fields])) AND ("8 bromo 1 methyl 6"[All Fields] AND "2-pyridinyl"[All Fields] AND "4h imidazo"[All Fields] AND ("1"[All Fields] AND "2-a"[All Fields]) AND ("1"[All Fields] AND "4"[All Fields]) AND "benzodiazepin-4-yl"[All Fields]) AND "propanoate"[All Fields]) OR "benzodiazepines"[MeSH Terms]) AND ("midazolam"[MeSH Terms] OR "midazolam"[All Fields] OR "versed"[All Fields] OR "flormidal"[All Fields] OR "dormicum"[All Fields] OR "hypnovel"[All Fields] OR ("roche"[All Fields] AND "midazolam"[All Fields]) AND ("bronchoscopy"[MeSH Terms] OR "bronchoscopy"[All Fields] OR "bronchoscopic procedure"[All Fields] OR ("bronchoscope"[All Fields] OR "bronchoscopes"[All Fields] OR "bronchoscoped"[All Fields] OR "bronchoscopic"[All Fields] OR "bronchoscopically"[All Fields]) OR ("endoscopy"[All Fields] AND "airway"[All Fields]) OR "airway endoscopy"[All Fields] OR ("pulmonary"[All Fields] AND "endoscopy"[All Fields]) OR "tracheobronchial endoscopy"[All Fields])) AND (clinicaltrial[Filter] OR randomizedcontrolledtrial[Filter]) | |
| **Our search strategy in WOS: (37)** | |
| **#1** | (remimazolam OR ONO 2745 OR ono2745 OR "ONO-2745" OR CNS 7056 OR methyl 38 bromo 1 methyl 6 2-pyridinyl 4h imidazol 2-a 1 4 benzodiazepin-4-yl propanoate OR Benzodiazepines) |
| **#2** | (midazolam OR Versed OR Dormicum OR Flormidal OR Hypnovel OR 8-chloro-6-(2-fluorophenyl)-1-methyl-4H-imidazo[1,5-a][1,4]benzodiazepine OR Roche Midazolam) |
| **#3** | (bronchoscopy OR bronchoscopic procedure OR bronchoscopic sedation OR bronchoscopes OR bronchoscopically OR bronchoscopic OR airway endoscopy OR pulmonary endoscopy OR tracheobronchial endoscopy OR bronchoscope) |
| **#4** | #3 AND #2 AND #1 |
| **Our search strategy in Embase:(28)** | |
| **1** | 'remimazolam'/exp OR remimazolam |
| **2** | 'cns7056' OR 'ono2745' OR 'ono 2745' |
| **3** | #1 OR #2 |
| **4** | 'midazolam'/exp |
| **5** | (versed OR dormicum OR flormidal OR hypnovel OR roche) AND midazolam) |
| **6** | #4 OR #5 |
| **7** | 'bronchoscopy'/exp |
| **8** | ((bronchoscopic AND procedure) OR (bronchoscopic AND sedation) OR bronchoscopes OR bronchoscopically OR bronchoscopic OR (airway AND endoscopy) OR (pulmonary AND endoscopy) OR (tracheobronchial AND endoscopy) OR bronchoscope) |
| **9** | #7 OR #8 |
| **10** | #3 AND #6 AND #9 |
| **Our search strategy in Cochrane: (37)** | |
| **1** | (remimazolam OR ONO 2745 OR ONO2745 OR CNS 7056 OR (methyl 38 bromo 1 methyl 6 2 pyridinyl 4h imidazo1 2 a 1 4 benzodiazepin 4 yl propanoate) OR Benzodiazepines) |
| **2** | bronchoscopy OR bronchoscopic procedure OR bronchoscopic sedation OR bronchoscopes OR bronchoscopically OR bronchoscopic OR airway endoscopy OR pulmonary endoscopy OR tracheobronchial endoscopy OR bronchoscope |
| **3** | midazolam OR Versed OR Dormicum OR Flormidal OR Hypnovel OR Roche Midazolam |
| **4** | MeSH descriptor: [Bronchoscopy] explode all trees |
| **5** | MeSH descriptor: [Midazolam] explode all trees |
| **6** | #2 OR #4 |
| **7** | #3 OR #5 |
| **8** | #1 AND #6 AND #7 |
| **scopus (137)** | |
| ( remimazolam OR ono 2745 OR ono2745 OR "ONO 2745" OR CNS 7056 OR benzodiazepines ) AND ( midazolam OR verses OR dormitum OR floridal OR hypnodil OR roche AND midazolam ) AND ( bronchoscopy OR bronchoscopic AND procedure OR bronchoscopic AND sedation OR bronchoscopes OR bronchoscopically OR bronchoscopic OR airway AND endoscopy OR pulmonary AND endoscopy OR tracheobronchial AND endoscopy OR bronchoscope ) | |
| **google scholar (138)** | |
| allintitle:(remimazolam OR ono2745 OR cns7056 OR "ono 2745" OR midazolam OR Versed OR Dormicum OR Flormidal OR Hypnovel OR "Roche Midazolam" OR Benzodiazepine OR sedative OR sedation OR anesthesia) (bronchoscopy OR bronchoscopic OR tracheobronchial OR bronchoscope) (RCT OR "Randomized Controlled Trial" OR "Random Allocation" OR "Randomized Experiment" OR "Controlled Clinical Trial" OR Randomized OR control OR "Double-Blind Trial" OR "Single-Blind Trial" OR "Open-Label Trial" OR "Placebo-Controlled Trial" OR "Parallel Group Trial" OR "Crossover Trial" OR "Factorial Trial" OR "Pragmatic Trial")allintitle:(remimazolam OR ono2745 OR cns7056 OR "ono 2745" OR midazolam OR Versed OR Dormicum OR Flormidal OR Hypnovel OR "Roche Midazolam" OR Benzodiazepine OR sedative OR sedation OR anesthesia) (bronchoscopy OR bronchoscopic OR tracheobronchial OR bronchoscope) (RCT OR "Randomized Controlled Trial" OR "Random Allocation" OR "Randomized Experiment" OR "Controlled Clinical Trial" OR Randomized OR control OR "Double-Blind Trial" OR "Single-Blind Trial" OR "Open-Label Trial" OR "Placebo-Controlled Trial" OR "Parallel Group Trial" OR "Crossover Trial" OR "Factorial Trial" OR "Pragmatic Trial")  —-----------  allintitle: (remimazolam OR ono2745 OR cns7056 OR "ono 2745") (midazolam OR Versed OR Dormicum OR Flormidal OR Hypnovel OR "Roche Midazolam") (bronchoscopy OR bronchoscopic OR tracheobronchial OR bronchoscope)  —-------------  allintitle: (remimazolam OR ono2745 OR cns7056 OR "ono 2745") (bronchoscopy OR bronchoscopic OR tracheobronchial OR bronchoscope) | |

**Studies and Participant’s Characteristics**

**Table S3: The number of patients with adverse events during bronchoscopy**

|  | Drug/Ref | Pastis et al[11], (2019) | Kim et al[12], (2023) | Huang et al[13], (2024) | Wu et al[14], (2024) |
| --- | --- | --- | --- | --- | --- |
| Patients in each group | Remimazolam | 303 | 49 | 34 | 46 |
|  | Midazolam | 69 | 51 | 30 | 48 |
| Hypoxia | Remimazolam | 66 | 1 | 7 | 4 |
|  | Midazolam | 13 | 1 | 3 | 4 |
| Hypotension | Remimazolam | 127 | 1 | NA | NA |
|  | Midazolam | 34 | 1 | NA | NA |
| Hypertension | Remimazolam | 186 | NA | 5 | NA |
|  | Midazolam | 41 | NA | 10 | NA |
| Tachycardia | Remimazolam | 4 | 2 | 1 | NA |
|  | Midazolam | 0 | 2 | 2 | NA |
| Cough | Remimazolam | NA | NA | 14 | 29 |
|  | Midazolam | NA | NA | 13 | 34 |
| Nausea | Remimazolam | 12 | NA | Na | 3 |
|  | Midazolam | 2 | NA | NA | 2 |
| Vomiting | Remimazolam | 6 | 0 | NA | 1 |
|  | Midazolam | 2 | 0 | NA | 0 |

NA: Not Applicable, Ref: Reference.

**Results of the meta-analysis**


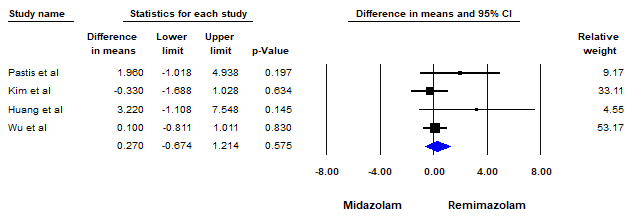


**Figure S1: Forest Plot for Comparison of Bronchoscopy Duration Between Remimazolam and Midazolam**

**
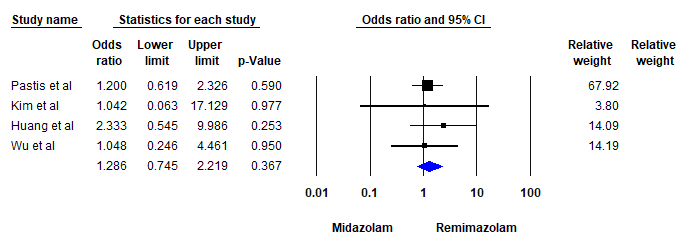
**

**Figure S2: Forest Plot for Comparison of Hypoxia Between Remimazolam and Midazolam**

**
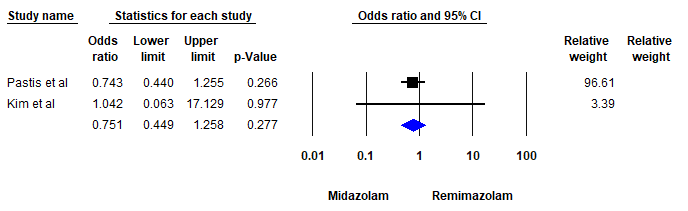
**

**Figure S3: Forest Plot for Comparison of Hypotension Incidence Between Remimazolam and Midazolam**

**
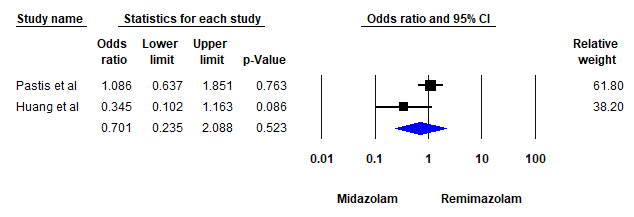
**

**Figure S4: Forest Plot for Comparison of Hypertension Incidence Between Remimazolam and Midazolam**

**
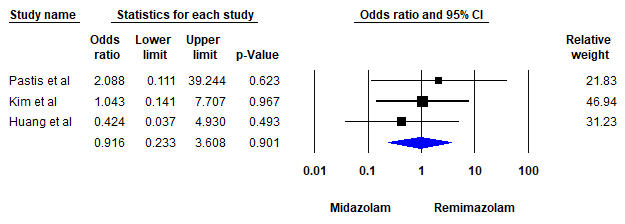
**

**Figure S5: Forest Plot for Comparison of Tachycardia Between Remimazolam and Midazolam**

**
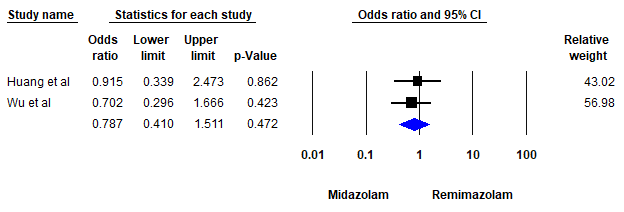
**

**Figure S6: Forest Plot for Comparison of Cough Incidence Between Remimazolam and Midazolam**

**
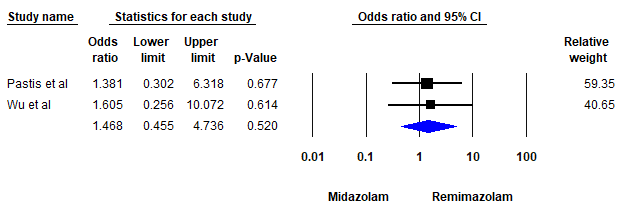
**

**Figure S7: Forest Plot for Comparison of Nausea Incidence Between Remimazolam and Midazolam**

**
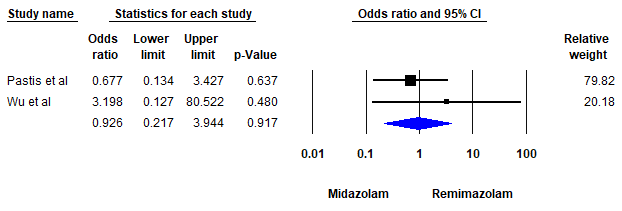
**

**Figure S8: Forest Plot for Comparison of Vomiting Incidence Between Remimazolam and Midazolam**

**Publication Bias**

**
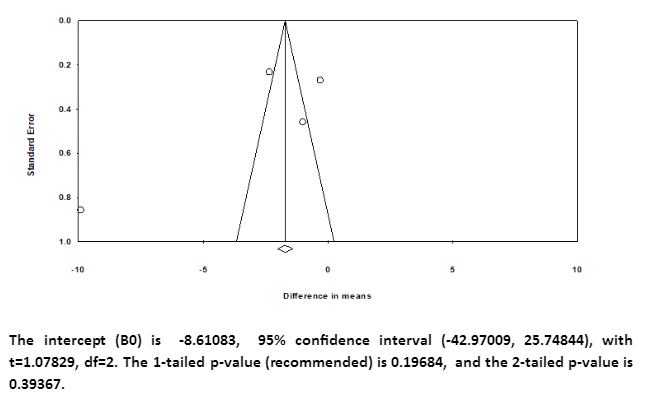
**

**Figure S9. Funnel Plot and Egger’s Regression Test Results for Comparison of Induction Time Between Remimazolam and Midazolam.**

**
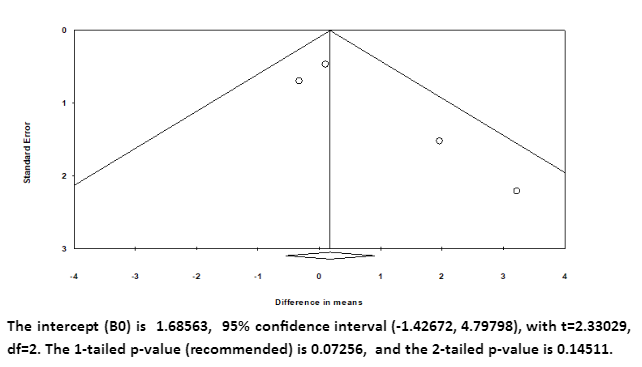
**

**Figure S10. Funnel Plot and Egger’s Regression Test Results for Comparison of Bronchoscopic Duration Between Remimazolam and Midazolam.**

**
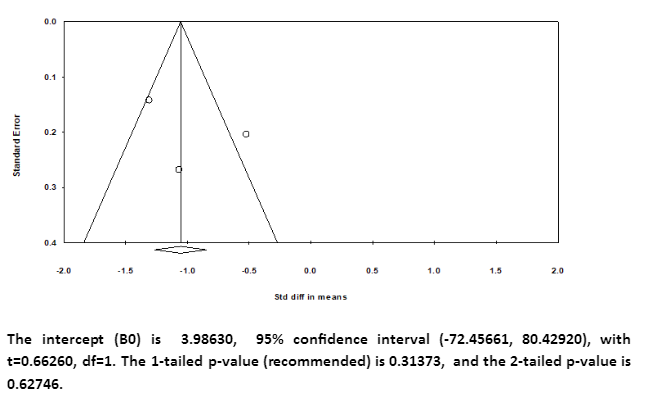
**

**Figure S11. Funnel Plot and Egger’s Regression Test Results for Comparison of Recovery Time Between Remimazolam and Midazolam.**

**
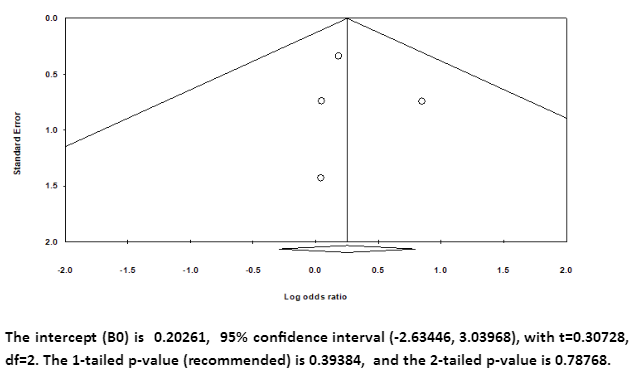
**

**Figure S12. Funnel Plot and Egger’s Regression Test Results for Comparison of Hypoxia Between Remimazolam and Midazolam.**

**
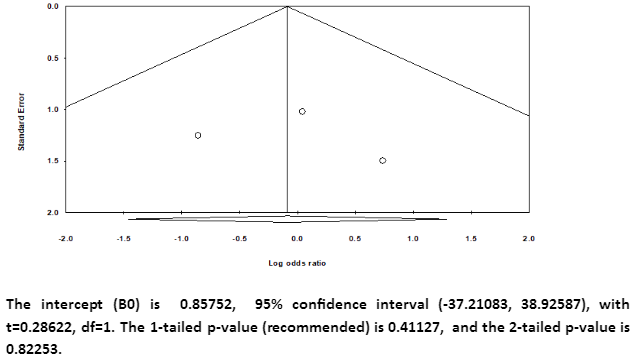
**

**Figure S13. Funnel Plot and Egger’s Regression Test Results for Comparison of Tachycardia Between Remimazolam and Midazolam.**

**Sensitivity analysis**

**
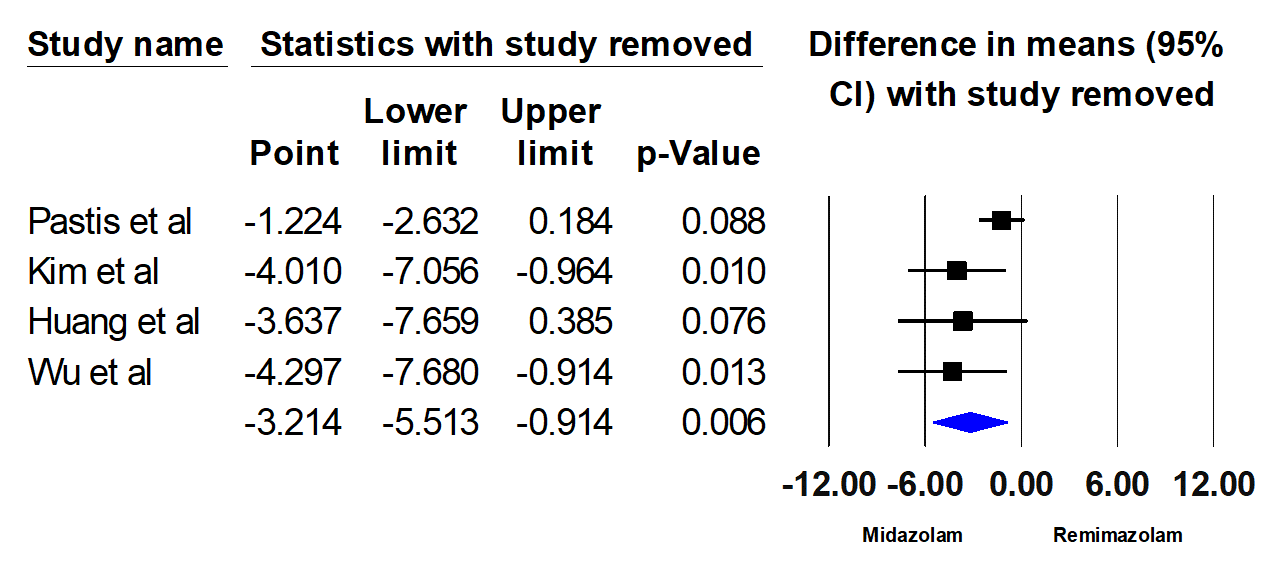
**

**Figure S14. Forest Plot with One Study Removed Sensitivity Analysis for Comparison of Induction Time Between Remimazolam and Midazolam.**

**
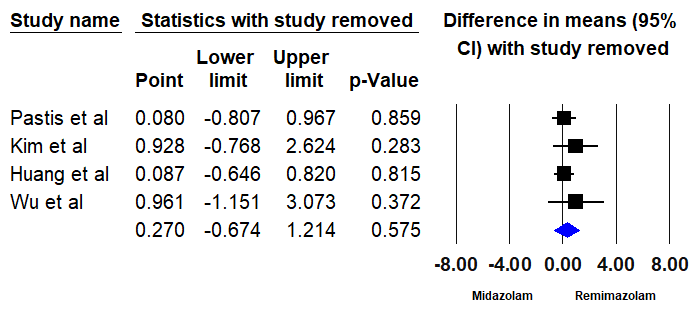
**

**Figure S15. Forest Plot with One Study Removed Sensitivity Analysis for Comparison of Bronchoscopic Duration Between Remimazolam and Midazolam.**

**
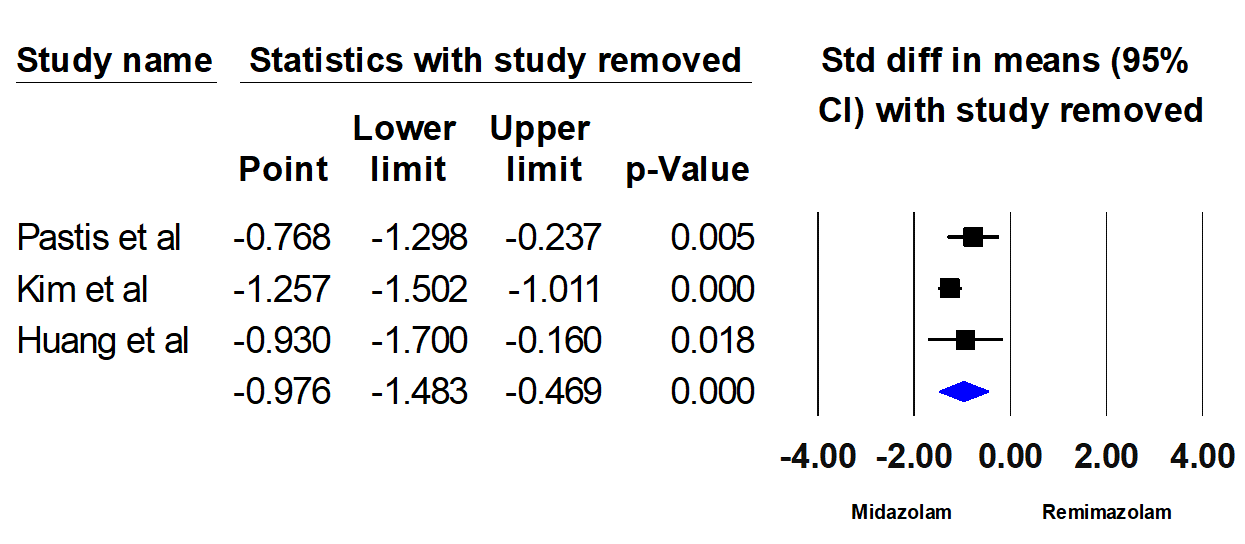
**

**Figure S16. Forest Plot with One Study Removed Sensitivity Analysis for Comparison of Recovery Time Between Remimazolam and Midazolam.**

**
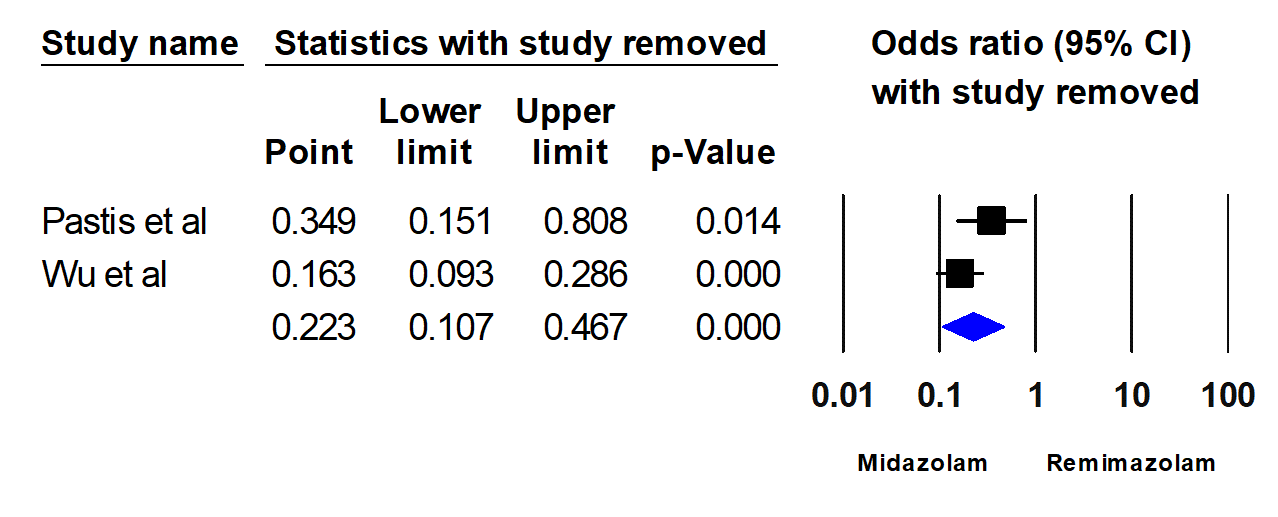
**

**Figure S17. Forest Plot with One Study Removed Sensitivity Analysis for Comparison of Administration of Rescue Sedatives Between Remimazolam and Midazolam.**

**
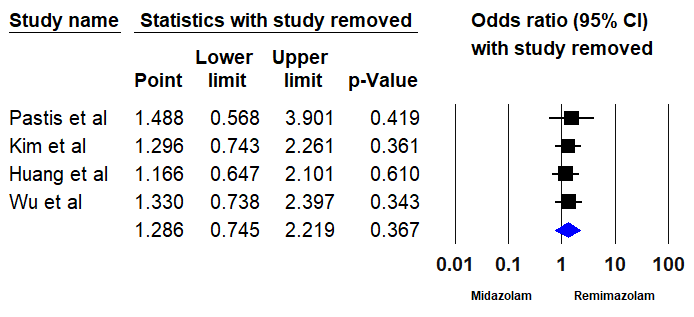
**

**Figure S18. Forest Plot with One Study Removed Sensitivity Analysis for Comparison of Hypoxia Between Remimazolam and Midazolam.**

**
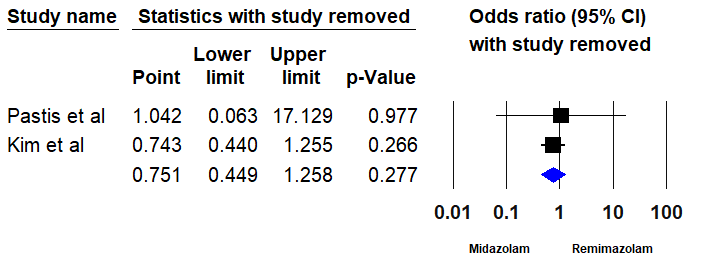
**

**Figure S19. Forest Plot with One Study Removed Sensitivity Analysis for Comparison of Hypotension Between Remimazolam and Midazolam.**

**
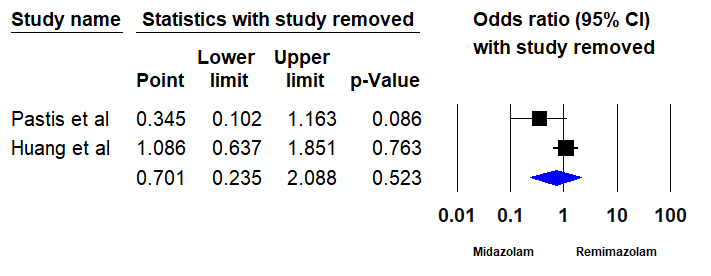
**

**Figure S20. Forest Plot with One Study Removed Sensitivity Analysis for Comparison of Hypertension Between Remimazolam and Midazolam.**

**
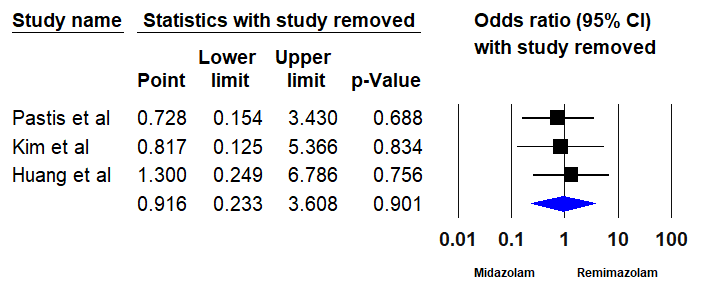
**

**Figure S21. Forest Plot with One Study Removed Sensitivity Analysis for Comparison of Tachycardia Between Remimazolam and Midazolam.**

**
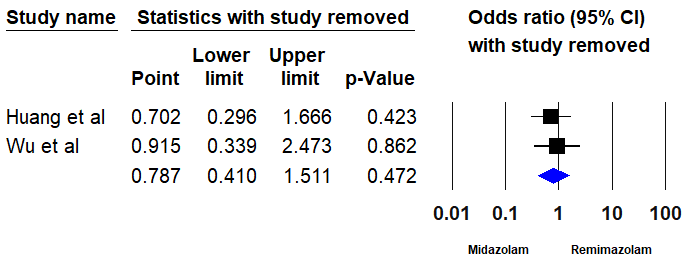
**

**Figure S22. Forest Plot with One Study Removed Sensitivity Analysis for Comparison of Cough Between Remimazolam and Midazolam.**

**
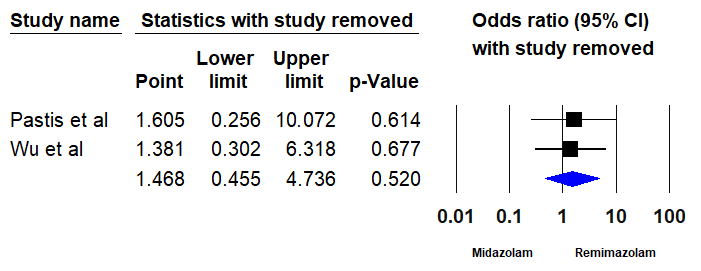
**

**Figure S23. Forest Plot with One Study Removed Sensitivity Analysis for Comparison of Nausea Between Remimazolam and Midazolam.**

**
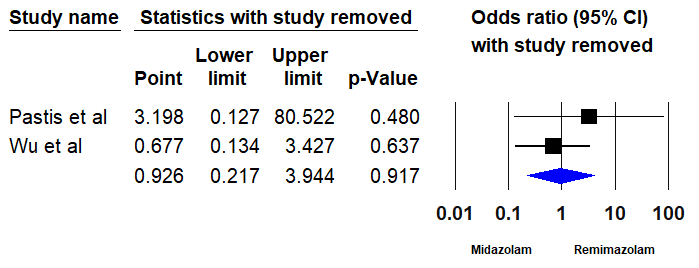
**

**Figure S24. Forest Plot with One Study Removed Sensitivity Analysis for Comparison of Vomiting Between Remimazolam and Midazolam.**
