# Supplementary material for: The comparison of remimazolam and midazolam in bronchoscopic sedation: a systematic review and meta-analysis of randomized controlled trials
Source: Front Med (Lausanne). 2025 Jul 8;12:1581280. doi: 10.3389/fmed.2025.1581280 (PMC12279844; doi:10.3389/fmed.2025.1581280)
Supplement: Supplementary file 2 [file Table_2.docx]

| **Table1: basic characteristics of included studies** | | | | | | | | | | | | | |
| --- | --- | --- | --- | --- | --- | --- | --- | --- | --- | --- | --- | --- | --- |
| Ref | Country | ASA  Status | Total  No | Remimazolam group | | | | | Midazolam group | | | | |
|  |  |  |  | No | Age mean (Y) | (n)  M/F | BMI | Dose | No | Age mean (Y) | (n) M/F | BMI | Dose |
| Pastis et al[15],  2019 | United  State | I-III | 372 | 303 | 62.7 ± 12.09 | 139/164 | 28.4 ± 6.39 | Initial dose: 5 mg and fentanyl 25 - 75 µg; Top-up dose: 2.5 mg midazolam for rescue | 69 | 61.5 ± 14.03 | 35/34 | 28 ± 5.79 | Initial dose: 1-1.75 mg and fentanyl 25 - 75 µg; Top-up dose: 0.5-1 mg midazolam for rescue |
| Kim et al[16],   2023 | South  Korea | I-III | 100 | 49 | 65 ± 14.07 | 31/  18 | 23.47 ± 3.44 | A<60 y or W>50 Kg : 5 mg,  A≥60 y or W<50 Kg : 3 mg; Top-up dose: 2.5 mg | 51 | 68 ± 11.11 | 30/21 | 21.9 ± 3.2 | A<60 y or W>50 Kg : 3 mg,  A≥60 y or W<50 Kg : 2 mg; Top-up dose: 0.5 mg |
| Huang et al[17],  2024 | China | I-II | 64 | 34 | 54.65 ± 13.23 | 22/  12 | 22.26 ± 3.65 | Initial dose: 0.2 mg/kg and fentanyl 0.5 µg/kg; Top-up dose: 25 µg fentanyl | 30 | 57.37 ± 12.36 | 18/12 | 23.2 ± 3.73 | Initial dose: 0.075mg/kg and fentanyl 0.5 µg/kg; Top-up dose: 25 µg fentanyl |
| Wu et al[18],  2024 | China | I-III | 94 | 46 | 70.37 ± 4.07 | 26/20 | 22.09 ± 3.65 | Initial dose: 0.135mg/kg and alfentanyl 18 µg/kg; pfopofol for rescue | 48 | 69.21 ± 3.59 | 29/19 | 22.19 ± 3.06 | Initial dose: 0.045mg/kg and alfentanyl 18 µg/kg; pfopofol for rescue |
| N: Number, ASA: American Society of Anesthesiologists, Y: Years, M: Male, F: Female, | | | | | | | | | | | | | |

| **Table 2: The summary of meta-analysis results for the comparison between Remimazolam and Midazolam for all out comes.** | | | | | | |  |  |
| --- | --- | --- | --- | --- | --- | --- | --- | --- |
| Outcome | No. of Patients | Effect size measure | Effect size | 95% CI | p-Value | Heterogeneity (I²) | Q Value | p-Value (Heterogeneity) |
| Induction Time (Min) | 630 | MD | -3.214 | (-5.513 to -0.914) | 0.006 | 97.666 | 128.523 | < 0.001 |
| Bronchoscopic Duration (Min) | 630 | MD | 0.270 | (-0.674 to 1.214) | 0.575 | 21.868 | 3.84 | 0.279 |
| Recovery Time (Min) | 536 | SMD | -0.976 | (-1.483 to -0.469) | < 0.001 | 80.118 | 10.06 | 0.007 |
| Rescue Sedative Use | 466 | OR | 0.223 | (0.107 to 0.467) | < 0.001 | 54.405 | 2.193 | 0.139 |
| Intraoperative Hypoxia | 630 | OR | 1.286 | (0.745 to 2.219) | 0.367 | < 0.001 | 0.786 | 0.853 |
| Intraoperative Hypotension | 473 | OR | 0.751 | (0.449 to 1.258) | 0.277 | < 0.001 | 0.054 | 0.816 |
| Intraoperative Hypertension | 436 | OR | 0.701 | (0.235 to 2.088) | 0.523 | 65.143 | 2.869 | 0.09 |
| Intraoperative Tachycardia | 536 | OR | 0.916 | (0.233 to 3.608) | 0.901 | < 0.001 | 0.698 | 0.706 |
| Intraoperative Cough | 158 | OR | 0.787 | (0.410 to 1.511) | 0.472 | < 0.001 | 0.155 | 0.693 |
| Postoperative Nausea | 466 | OR | 1.468 | (0.455 to 4.736) | 0.52 | < 0.001 | 0.015 | 0.902 |
| Postoperative Vomiting | 466 | OR | 0.926 | (0.217 to 3.944) | 0.917 | < 0.001 | 0.71 | 0.399 |
| OR: Odds ratio, Min: Minutes, MD: Mean difference, SMD: Standardized mean difference, No: Number. | | | | | | | |  |

| **Table 3: Summary of Outcomes and Certainty of evidence based on the Grading of Recommendations Assessment, Development and Evaluation (GRADE) approach** | | | | | | | | | |
| --- | --- | --- | --- | --- | --- | --- | --- | --- | --- |
| **Outcome** | **Number of Studies** | **Risk of Bias** | **Inconsistency** | **Indirectness** | **Imprecision** | No of patients | | | |
|  |  |  |  |  |  | **Remimazolam** | **Midazilam** | **Effect Estimate**  **(95% CI)** | **Grade** |
| Induction Time | 4 [15,16,17,18] | Low | Serious  (I² = 97.666),b | Not Serious | Serious | 432 | 198 | -3.214 minutes (-5.513 to -0.914) | Low ⨁ ⨁ ◯ ◯ |
| Bronchoscopic Duration | 4 [15,16,17,18] | Low | Not Serious | Not Serious | Not Serious | 432 | 198 | 0.270 minutes (-0.674 to 1.214) | High ⨁ ⨁ ⨁ ⨁ |
| Recovery Time | 3 [15,16,17] | Low | Moderate (I² = 80.118),b | Not Serious | Not Serious | 129 | 129 | -0.976 (-1.483 to -0.469) | High ⨁ ⨁ ⨁ ⨁ |
| Rescue Sedative Use | 2 [15,18] | Low | Moderate  (I² = 54.405),b | Not Serious | Not Serious | 64/349 | 66/117 | 0.223 (0.107 to 0.467) | High ⨁ ⨁ ⨁ ⨁ |
| Hypoxia | 4  [15,16,17,18] | Low | Not Serious | Not Serious | Not Serious | 78/432 | 21/198 | 1.286 (0.745 to 2.219) | High ⨁ ⨁ ⨁ ⨁ |
| Hypotension | 2 [15,16] | Low | Not Serious | Not Serious | Not Serious | 128/352 | 35/120 | 0.751 (0.449 to 1.258) | High ⨁ ⨁ ⨁ ⨁ |
| Hypertension | 2 [15,17] | Low | Moderate  (I² = 65.143),b | Not Serious | Serious | 191/337 | 51/99 | 0.701 (0.235 to 2.088) | Moderate ⨁ ⨁ ⨁ ◯ |
| Tachycardia | 3 [15,16,17] | Low | Not Serious | Not Serious | Serious | 7/386 | 4/150 | 0.916 (0.233 to 3.608) | Moderate ⨁ ⨁ ⨁ ◯ |
| Cough | 2 [17,18] | Some Concern | Not Serious | Not Serious | Serious | 43/80 | 47/78 | 0.787 (0.410 to 1.511) | Low ⨁ ⨁ ◯ ◯ |
| Postoperative Nausea | 2 [15,18] | Low | Not Serious | Not Serious | Serious | 15/349 | 4/117 | 1.468 (0.455 to 4.736) | Moderate ⨁ ⨁ ⨁ ◯ |
| Postoperative Vomiting | 2  [15,18] | Low | Not Serious | Not Serious | Serious | 7/398 | 2/168 | 0.926 (0.217 to 3.944) | Moderate ⨁ ⨁ ⨁ ◯ |
| Cl: confidence interval, a. The proportion of information from studies at high risk of bias is sufficient to affect the interpretation  of results, b. I suggested considerable heterogeneity, c. For comparison of the incidence of rare events, the total sample size  appeared insufficient, and the 95% Cl was too wide. | | | | | | | | | |
|  |  |  |  |  |  |  |  |  |  |
|  |  |  |  |  |  |  |  |  |  |
